# Regular Physical Activity Can Counteract LONG COVID Symptoms in Adults over 40

**DOI:** 10.3390/jfmk9030119

**Published:** 2024-07-04

**Authors:** Marco Centorbi, Giulia Di Martino, Carlo della Valle, Enzo Iuliano, Gloria Di Claudio, Amelia Mascioli, Giuseppe Calcagno, Alessandra di Cagno, Andrea Buonsenso, Giovanni Fiorilli

**Affiliations:** 1 Department of Medicine and Health Sciences, University of Molise, 86100 Campobasso, Italy; marco.centorbi@hotmail.it (M.C.); giulia.dimartino21@gmail.com (G.D.M.); diclaudiogloria@gmail.com (G.D.C.); amelia.mascioli@unimol.it (A.M.); giuseppe.calcagno@unimol.it (G.C.); andrea.buonsenso@unimol.it (A.B.); fiorilli@unimol.it (G.F.); 2 Department of Neurosciences, Biomedicine and Movement, University of Verona, 37134 Verona, Italy; carlo.dellavalle@univr.it; 3 Faculty of Psychology, eCampus University, 22060 Novedrate, Italy; enzo.iuliano@uniecampus.it; 4 Department of Movement, Human and Health Sciences, University of Rome “Foro Italico”, 00135 Rome, Italy; 5 Department of Human Sciences, Guglielmo Marconi University, Via Plinio 44, 00193 Rome, Italy

**Keywords:** exercise, fatigue, distress, pandemic

## Abstract

Three years after the SARS-CoV-19 pandemic, a chronic post-COVID syndrome “LONG COVID” persists, causing fatigue and shortness of breath, along with distress, anxiety, and depression. Aim: To assess the impact of physical activity on the management and rehabilitation of LONG COVID, as well as to investigate the persistence of LONG COVID symptomatology in individuals over 40 years, beyond the pandemic. Methods: A total of 1004 participants (aged 53.45 ± 11.35) were recruited through an online snowball sampling strategy to complete a web-based survey. The following questionnaires were administered: Physical Activity Scale for Elderly (PASE), Shortness of Breath Questionnaire (SOBQ), Patient Health Questionnaire-9 item (PHQ-9), Generalized Anxiety Disorder 7-item (GAD-7), and Fatigue Scale for Motor and Cognitive Functions (FSMC). Results: Significant gender differences were discovered, with women reporting higher symptoms than men (*p* < 0.001). Significant age differences were also found, with participants under 55 showing higher values than those over 55 (*p* < 0.001). No significant differences were found between aerobic and mixed physical activity (*p* > 0.05) while significant results emerged between physical activity groups and the no activity group (*p* < 0.001). The low-frequency group reported higher symptoms than the high-frequency group (all *ps* < 0.001). Conclusion: Regardless of the type of physical activity performed, our survey identified the frequency of training as a crucial factor to overcome LONG COVID symptoms; the challenge lies in overcoming the difficulties due to the persistent feelings of inefficiency and fatigue typical of those who have contracted the infection.

## 1. Introduction

The coronavirus disease 2019 (COVID-19) has presented unprecedented challenges to global healthcare and public health systems, leading to restrictions in Italy and worldwide [1]. Despite the decline in infection cases, the enduring repercussions of SARS-CoV-2 infection remain significant. Three years after the SARS-CoV-2 pandemic, a chronic post-COVID syndrome known as “LONG COVID” persists, characterized by somatic manifestations such as fatigue and dyspnoea, alongside affective and psychological symptoms like distress, anxiety, and depression [2]. Recently, the World Health Organization (WHO) [3] defined it “as the continuation or development of new symptoms 3 months after the initial SARS-CoV-2 infection, with these symptoms lasting for at least 2 months with no other explanation”.

LONG COVID affected 17 million people in Europe during the acute phase and an estimated 65 million people globally [4]. A report from Italy highlighted that 87% of individuals experienced the persistence of at least one symptom [5].

Interventions developed to address these physical and psychological disorders underscore interdisciplinary approaches based on social interaction, communication, and physical fitness, which could aid in overcoming the disease [6]. Particularly against depression and anxiety, physical activity (PA), characterized by higher volume loads and frequency, has been recognized as a protective factor [7]. Moreover, maintaining a stable routine of PA and high adherence to protocols could facilitate stable improvements [8].

Meira et al. [9] found that there was a notable difference when comparing the group that engaged in physical activity for 40 min or more with the group that exercised for less than 40 min. The results were more pronounced for the group that exercised for at least 40 min on three or more days per week compared to those who exercised less or did not exercise at all. Even more remarkable were the results for the group that exercised for at least 40 min on five or more days per week compared to those who exercised for less than three days a week for 40 min. These findings appear to give evidence that not “any” or “general” physical activity was capable of lessening anxiety. Thus, the frequency and duration of physical activity seems to play a pivotal role in reducing anxiety levels during the COVID-19 pandemic.

Moreover, de Camargo [10] found that people who engaged in PA for 30 min, four to five days a week, showed a lower level of stress correlated to SARS-CoV-2.

Given that engagement in PA before, during, and after SARS-CoV-2 spread appears to significantly influence three critical outcomes post-COVID-19 (hospitalization, ICU admission, and mortality), several researchers demonstrated that PA, or rather familiarity with PA, could be a beneficial predictive element in relieving symptoms associated with long-term COVID-19 [11].

The purpose of this survey was to assess the impact of PA on the management and rehabilitation of LONG COVID, as well as to investigate the persistence of LONG COVID symptomatology in individuals aged 40 years and older, beyond the pandemic. The research questions revolved around whether the symptomatology of LONG COVID could be definitively overcome through PA and if engagement in PA provided protection against the persistence of these symptoms.

## 2. Materials and Methods

### 2.1. Study Design

The study is a retrospective cohort study designed to investigate whether engaging in PA could represent a protective factor against symptoms of LONG COVID in an over 40 adult population. For this reason, the survey aimed to evaluate whether physical and psychological symptoms were widespread in both the physically active and no activity population.

### 2.2. Participants

A total of 1004 participants (aged 53.4 ± 11.3) were included in this study. Participants were recruited through an online snowball sampling strategy and were asked to complete a web-based survey, to obtain physical and psychological information concerning the LONG COVID symptoms. The G*Power software (v.3.1.9.6) was used to calculated a priori the minimum sample size. A minimum sample size of 148 participants was necessary to have a moderate effect size f = 0.30. The inclusion criteria were as follows: (1) to be aged over 40 years [12]; (2) having contracted COVID-19; (3) having experienced COVID positivity for at least 14 days with and without symptoms. The exclusion criteria were as follows: (1) having chronic or acute respiratory, metabolic, or cardiovascular disease before contracting COVID-19; (2) having experienced injuries or surgeries in the last year that could have limited the potential engagement in PA. Incomplete questionnaires or those filled out by subjects who did not meet the inclusion criteria were discarded. We used as the cut-off for eliminating incomplete questionnaires the non-completion of at least 3 questions. Before completing the questionnaire, a cover letter was administered to inform the participants about the nature and purpose of the study, as well as the anonymity procedures. In addition, electronic informed consent was obtained from all participants. The study was designed and conducted in accordance with the Declaration of Helsinki and approved by the Scientific Technical Committee of the Department of Medicine and Health Sciences, University of Molise (Prot. n. 05/2022).

### 2.3. Procedures

The survey was administered through an online platform (Google Form, Google, Mountain View, CA, USA) from November 2023 to March 2024. The Section 1 aimed to obtain information regarding sociodemographic data such as age and gender, and regarding the practice and the level of PA (active and not active, with the cut-off indicated by WHO guidelines), its primary metabolic demand (aerobic, mixed anaerobic, and aerobic, and not active) in accordance with the classification proposed by Dal Monte (2000) [13], the weekly training frequency (active low frequency, active high frequency), for how many years they have been engaged in PA (0, 1–3 years, 4–7 years, and more than 7 years), the duration of symptoms related to COVID-19 infection (no symptoms, few days, almost 4 weeks, several months), and the severity of COVID-19 infection (slight, moderate, and severe). The frequency group was determined according to the weekly training frequency of each participant. The low-frequency group included participants with less than 3 PA sessions per week, while the high-frequency group included participants with 3 or more PA sessions per week. The training experience identified how many years the interviewed subjects had been regularly engaging in structured sports or physical activities. The severity of COVID-19 infection was established as follows: participants with no symptoms and those who did not seek medical intervention were categorized as having slight severity; participants requiring medical intervention without hospitalization were classified as moderate severity; hospitalized participants were classified as severe cases. Participants were as follows: 634 active adults (age 53.7 ± 11.5) and 370 not active adults (age 53.0 ± 11.2) including both men (*n* = 322) and women (*n* = 682). The sample characteristics are shown in Table 1.

In the Section 2, the following questionnaires were administered to quantify the level of activities of daily living (ADLs) through the Physical Activity Scale for Elderly (PASE) [14], and the symptoms related to the LONG COVID condition: Shortness of Breath Questionnaire (SOBQ) [15], Patient Health Questionnaire-9 item (PHQ-9) [16], Generalized Anxiety Disorder 7-item (GAD-7) [17], and Fatigue Scale for Motor and Cognitive Functions (FSMC) [18].

*Physical Activity Scale for Elderly* (*PASE*): This questionnaire assesses ADLs through 12 self-administered items. It evaluates three categories of PA: leisure time, household, and work activities. Scores range from 0 to 793, with a higher score indicating a higher level of weekly PA [19].

*Shortness of Breath Questionnaire* (*SOBQ*): The questionnaire was designed to assess the shortness of breath during different daily living activities. In particular, participants were required to indicate how much shortness of breath affects 21 different daily activities on a 6-point scale (0 = not at all, 5 = unable to do). In addition, three supplementary questions related to shortness of breath, fear of harm from overexertion, and fear of shortness of breath have been added. If patients do not regularly engage in the activity, they are requested to estimate the expected degree of shortness of breath. The SOBQ is scored by summing responses across all 24 items to generate a total score ranging from 0 to 120, with higher scores indicating greater shortness of breath.

*Patient Health Questionnaire-9 item* (*PHQ-9*): This is a self-report questionnaire to evaluate depressive disorder. Participants were asked to indicate for each of the 9 items how much a symptom occurs on a scale from 0 to 3 (0 = not at all, 1 = several days, 2 = more than half of the days, 3 = nearly every day). The total score ranges from 0 to 27, with a higher score indicating major depressive disorder. The total score was split as follows: “no disorder” (from 0 to 9), “mild disorder” (from 10 to 14), “moderate disorder” (from 15 to 19), and “severe disorder” (≥20).

*Generalized Anxiety Disorder 7-item* (*GAD-7*): This is a self-report questionnaire to evaluate generalized anxiety symptoms. The questionnaire explores 7 core symptoms: nervousness, inability to stop worrying, excessive worry, restlessness, difficulty in relaxing, irritation, and fear of something happening. Participants were asked to rate from 0 to 3 each statement (0 = not at all, 1 = several days, 2 = more than half of the days, 3 = nearly every day). The total score ranges from 0 to 21, with a higher score indicating major anxiety disorder. A total score ≤ 4 indicates no disorder, a score from 5 to 9 indicates mild anxiety, a score from 10 to 14 indicates moderate anxiety, and a score ≥ 15 indicates severe anxiety.

*Fatigue Scale for Motor and Cognitive Functions* (*FSMC*): The questionnaire evaluates the subjective feeling of motor and cognitive aspects of fatigue. It consists of 20 items equally divided for the motor and cognitive aspects. Participants were asked to rate from 1 “does not apply at all” to 5 “applies completely” each statement related to everyday life. The total score ranges from 20 to 100, with a higher score indicating major fatigue symptoms. The total score was split as follows: a total score ≥ 43 indicates mild fatigue, ≥53 indicates moderate fatigue, and ≥63 indicates severe fatigue. A cognitive subscale score ≥ 22 indicates mild, ≥28 indicates moderate, and ≥34 indicates severe cognitive fatigue. For the motor subscale, a score ≥ 22 indicates mild, ≥27 indicates moderate, and ≥32 indicates severe motor fatigue.

### 2.4. Statistical Analysis

Data analysis was performed using SPSS Statistics 23 (IBM) software. Descriptive statistics are presented as mean and standard deviations (SD). The normal distribution of data was assessed using the Kolmogorov–Smirnov test. Data were not normally distributed, so a non-parametric statistical approach was used through the Kruskal–Wallis test to evaluate differences among SOBQ, PHQ-9, GAD-7, and FSMC as dependent variables and age (under 55 and over 55), gender, primary metabolic demand (aerobic, mixed aerobic and anaerobic, not active), PA frequency (active low frequency, active high frequency, not active), training experience (0 years, 1–3 years, 4–7 years, more than 7 years), and COVID-19 symptoms duration (no symptoms, few days, 1 month, some months) as independent variables. Post hoc comparisons were performed using the U test of Mann–Whitney and the Bonferroni alpha level correction was applied. The alpha test level for statistical significance for all variables was set at 0.05. In addition, a Spearman rho correlation was performed to assess whether there was a relationship between PACES and/or training experience and the administered questionnaire. For the interpretation of the Spearman rho results, the following ranges were considered: values less than 0.3 were considered as a weak correlation; values between 0.3 and 0.7 were considered as a moderate correlation; values greater than 0.7 were considered as a strong correlation [20]. Finally, multiple linear regression analysis was performed to select the best predictors of all symptoms analysed among the ADLs and training experience. Due to the possibility of a correlation between PASE score and training experience, the variance inflation factor (VIF) was calculated to assess the validity of the analysis.

## 3. Results

The Kruskal–Wallis analysis showed significant differences for gender, where females reported higher symptoms than males for all the analysed questionnaires (*p* < 0.001).

Significant differences were found for age, where the under 55 group showed higher values than the over 55 group for all questionnaires (*p* < 0.001), except for SOBQ (*p* = 0.436).

No significant differences were found between aerobic and mixed physical activities while significant results were found between both the aerobic and mixed physical activity groups and the not active group for all symptoms (*p* < 0.001).

Significant differences were found for PA activity frequency, where both the low-frequency and high-frequency activity groups reported lower symptoms than not active group (all *ps* < 0.01) for all questionnaires. In addition, the low-frequency group reported higher symptoms than the high-frequency group (all *ps* < 0.001).

Regarding training experience, significant differences were found for SOBQ, where participants with no training experiences reported higher values than participant with more than 4 years of experience (*p* < 0.01) and participants with more than 7 years of experience reported lower values than those with less than 4–7 years (*p* < 0.05). Significant differences were found for PHQ-9, where participants with no training experience reported higher values than participants with more than 4 years of experience (*p* < 0.01) and participants with more than 7 years of experience reported lower values than those with less than 3 years (*p* < 0.001). Regarding GAD-7, participants with no training experience reported higher values than participants with more than 1 year of experience (*p* < 0.01) and participants with more than 7 years of experience reported lower values than those with less than 7 years (*p* < 0.05).

Finally, regarding COVID-19 symptom duration, groups with longer symptom duration during COVID-19 infection reported higher values compared to the groups with shorter duration (all *ps* < 0.01), except for GAD-7 comparing the no symptoms group with the few days group. All the results regarding SOBQ, PHQ-9, and GAD-7 are reported in Table 2a.

Finally, regarding FSMC and its subscales, participants with no training experience reported higher values than participants with more than 4 years of experience (*p* < 0.001) and participants with 1–3 years of experience reported higher values than those with more than 4 years (*p* < 0.01). The FSMC results are shown in Table 2b.

The Spearman *rho* correlation showed a weak inverse correlation (*rho* < 0.3, *p* < 0.001) between training experience and the administered questionnaires, including ADLs, and a moderate inverse correlation with GAD-7 (*rho* = −0.32, *p* < 0.001). Participants with higher training experience tend to report less LONG COVID symptoms. No significant correlation was found between ADLs and the administered questionnaires. The results of the correlation analysis are shown in Table 3.

The multiple linear regression analysis showed that the best predictor of all symptoms analysed was training experience (R^2^ between 0.039 and 0.057; *p* < 0.001). The VIF analysis showed that PASE score and training experience had a VIF value of 1, indicating the validity of the regression analysis. The coefficients of the multiple linear model are presented in Table 4.

## 4. Discussion

The data from this survey confirmed the hypothesis of this study: individuals over 40 years who engage in regular PA are better protected against the post-acute symptoms of SARS-CoV-2 infection, commonly referred to as LONG COVID. Regularly engaging in PA is a cost-effective and optimal strategy for all individuals to achieve physical and cognitive benefits [21] and provides some coping strategies [22]. Recently, a new theory has been formulated that conceptualizes LONG COVID as a phenomenon, including both physiological disease symptoms and physio-somatic and affective symptoms, that can interact with each other, thereby creating a comprehensive syndrome.

The majority of the population interviewed in this study (97.6%) experienced mild symptoms during COVID-19 infection. However, Bai and colleagues [23] found LONG COVID symptoms even in subjects who reported a mild COVID-19 infection. This characteristic may depend on the study design, which identified active subjects at various levels as the inclusion criterion.

No significant correlation was found between the participants’ level of daily activity (PASE) and their levels of anxiety, depression, stress, fatigue, and shortness of breath, as assessed by the questionnaires. Indeed, it should be noted that PASE assesses only the activities of daily living (ADLs) rather than the amount of PA practiced. Nevertheless, the moderate inverse correlation between years of physical activity training (experience) and symptoms such as anxiety, depression, and other typical LONG COVID symptoms, highlights the importance of consistently engaging in regular PA over the long term [22,24]. This correlation suggests that if PA is practiced continuously, in this case for more than 4 years, it may play a protective role against the symptoms of LONG COVID. Moreover, active behaviours and PA probably provide the active participants a sense of normality in practicing their daily routine again [25]. PA could represent an investment in terms of feeling normal [26]. Moreover, participants for whom PA is a core part of their weekly routine recognized PA as a strong motivator to improve their functional capacities. It is well known that exercise improves cardiorespiratory efficiency [27]. This helped them to overcome their sense of fatigue, which is one of the primary symptoms of LONG COVID, otherwise worsened by their emotional state.

Age, gender, pre-existing medical conditions, active lifestyle, and notably, psychological and stress-related factors have been identified as influencing the severity and duration of its symptoms [28,29].

Comparing the groups of interviewed participants, according to their level and duration of PA performed, the results highlighted that long-term activity significantly protected against symptoms of fatigue, anxiety, and depression [30]. Physically active individuals also exhibit a significantly lower physiological index of breathlessness difficulty compared to not active individuals. These findings are consistent with previous studies [31,32]. It should be noted that the physical and psychological effects of LONG COVID are often interconnected. Emotional implications and frustrations arise from the debilitating sensations, fatigue, and mental fog, making daily tasks challenging. This condition increases psychological distress, leading to anxiety and depression [33].

Comparing the different groups according to age, it was observed that subjects under 55 exhibit higher levels of cognitive and physical fatigue, anxiety, and depression than those over 55, as persistent symptoms after full recovery from coronavirus disease. Despite the expectation that older individuals would exhibit higher levels of fatigue, muscle pain, and respiratory disorders due to a lower capacity for recovery and greater organ function decline [23,34,35], as well as higher levels of anxiety and depression [36], it is probable that those under 55 were less accustomed to coping with the symptoms characteristic of LONG COVID, possibly because they have developed fewer coping abilities due to their better health condition [37,38]. Another possible explanation could be linked to the fact that the under 55 population in our study reported both lower PASE levels and fewer years of training experience compared to the over 55 population. The participants under 55 in this study were less active than the over 55 ones. It is likely they have had more free time than those under 55 to dedicate to PA [39]. Considering that regular and moderate PA, performed more by older responders, is a useful non-pharmacological intervention suitable for reducing the levels of distress and physiological and psychological distress factors, such as anxiety and depression that predispose to LONG COVID symptoms or exacerbate its severity, the older participants were more protected than those under 55 [40].

Women reported more symptoms of LONG COVID than men. This is in line with previous findings, that reported that women have a 3-fold higher risk of experiencing LONG COVID symptomatology [23,41,42]. However, there are conflicting data regarding the association between women and the LONG COVID condition. Several studies showed the prevalence of fatigue and other related symptoms in women [34,43], while other authors found no gender association [5,44]. Females showed lower resiliency and higher perceived stress perception, also related to life events. Resilience resulted to be strongly correlated with levels of PA in males compared to females [45]. Nevertheless, females show a higher level of avoidance than males which is a psychological defence to actively remove unpleasant thoughts [46].

Participants with longer symptom duration during COVID-19 infection reported higher LONG COVID symptoms compared to the those with shorter duration. This may be viewed as a psychosomatic response resulting from the prolonged trauma of having contracted the virus for an extended period [46].

Significant differences between active and not active individuals were found, without differences among types of PA, such as aerobic and mixed. A key determinant was the frequency of training. Those who engaged in PA at least three times a week reported significantly fewer symptoms compared to those who engaged in it less than three times a week and, naturally, compared to the not active population. Training frequency and regularity in performing PA, as confirmed by other studies, are fundamentally important in preserving both physical and mental health.

The limitations of this study are listed as follows. Firstly, the symptomatology and level of PA were subjectively and retrospectively assessed, which could raise concerns about memory bias. Secondly, the participants were only interviewed about some of the LONG COVID symptoms, whereas we know there are many more. Thirdly, the interviewed sample consisted of a higher number of women compared to men. Another limitation of the study was the inability to retrospectively obtain information on nutritional aspects such as food intake patterns and anti-inflammatory intake.

## 5. Conclusions

In conclusion, it is essential to remain as active as possible and maintain regular exercise to counteract the complex post-COVID symptomatology. This approach confirms the widely recognized beneficial impact of PA on both physical and mental health [47]. The challenge lies in overcoming the difficulties due to the persistent feelings of inefficiency and fatigue typical of those who have contracted the infection, which seem to last for an extended period or overcoming these difficulties could be related to less free time [48]. Another crucial protective factor provided by PA against LONG COVID psychological issues is its ability to ensure frequent social contacts over time [49]. Particular attention should be given to issues related to women, who have lower resilience to traumatic events.

Future research could verify whether different types of physical activity, from a physiological perspective, may produce varying protective effects. Additionally, it would be interesting to examine the combined effects of these and other LONG COVID symptoms to identify optimal intervention strategies.

## Figures and Tables

**Table 1:** Sample characteristics.

Variable	*n* (%)	PASE	Training Experience (Years)
**Total**	1004 (100%)	102.6 ± 50.5	9.9 ± 12.1
**Age (mean ± SD)**	53.4 ± 11.3		
Under 55	597 (59.5%)	99.2 ± 49.5	8.9 ± 11.2
Over 55	407 (40.5%)	107.7 ± 51.6	11.4 ± 13.1
**Gender**			
Male	322 (32.1%)	96.1 ± 55.1	13.4 ± 12.9
Female	682 (67.9%)	105.7 ± 47.9	8.3 ± 11.3
**Primary metabolic demand**			
Aerobic	417 (41.5%)	111.4 ± 51.6	15.4 ± 11.7
Mixed	217 (21.6%)	102. 6 ± 51.1	16.5 ± 12.1
Not active	370 (36.9%)	92.8 ± 47.1	0
**Physical activity frequency**			
Active low frequency	265 (26.4%)	100.7 ± 49.9	13.3 ± 11.4
Active high frequency	369 (36.7%)	113.9 ± 52.1	17.5 ± 11.8
Not active	370 (36.9%)	92.8 ± 47.1	0
**Training experience**			
0 years	370 (36.9%)	92.8 ± 47.1	0
1–3 years	146 (14.5%)	101.0 ± 49.3	1.8 ± 0.8
4–7 years	112 (11.2%)	104.5 ± 51.5	5.6 ± 1.1
More than 7 years	376 (37.4%)	112.4 ± 52.2	24.2 ± 7.5
**COVID-19 symptoms duration**			
No symptoms	223 (22.2%)	105.7 ± 60.6	10.0 ± 12.8
Few days	433 (43.1%)	103.0 ± 49.8	11.8 ± 12.2
1 month	220 (21.9%)	99.5 ± 43.8	8.3 ± 11.6
Some months	128 (12.8%)	101.3 ± 50.5	6.4 ± 9.9
**COVID-19 infection severity**			
Slight	980 (97.6%)	102.8 ± 50.5	10. 1 ± 12.1
Moderate	19 (1.9%)	103.5 ± 49.0	2.8 ± 6.3
Severe	5 (0.5%)	66.1 ± 49.3	0.4 ± 0.9

**Table 2:** (**a**). Significant differences between groups for SOBQ, PHQ-9, and GAD-7. Data are presented as mean ± standard deviation. (**b**). Significant differences between groups for FSMC and its subscales. Data are presented as mean ± standard deviation.

**(a)**				
**Categories**	**Groups**	**SOBQ**	**PHQ-9**	**GAD-7**
**Gender**	Male	35.2 ± 17.1	8.9 ± 6.5	7.4 ± 4.2
	Female	38.8 ± 17.9	11.7 ± 7.1	8.8 ± 4.8
***p*** **-value**	Male–Female	*p* < 0.001 *	*p* < 0.001 *	*p* < 0.001 *
**Age**	Under 55	37.7 ± 17.2	11.6 ± 7.1	8.8 ± 4.8
	Over 55	37.5 ± 18.4	9.7 ± 7.0	7.7 ± 4.4
***p*** **-value**	Under 55–Over 55	*p* = 0.436	*p* < 0.001 *	*p* < 0.001 *
**Primary metabolic demand**	Aerobic	35.3 ± 15.4	9.6 ± 6.1	7.5 ± 4.2
	Mixed	34.2 ± 13.6	9.7 ± 6.4	7.4 ± 3.9
	Not active	42.3 ± 21.0	12.8 ± 8.0	9.8 ± 5.2
***p*** **-value**	Aerobic–Mixed	*p* = 0.147	*p* = 0.985	*p* = 0.713
	Aerobic–Not active	*p* < 0.001 *	*p* < 0.001 *	*p* < 0.001 *
	Mixed–Not active	*p* < 0.001 *	*p* < 0.001 *	*p* < 0.001 *
**Physical activity frequency**	Active LF	36.5 ± 15.8	10.5 ± 6.1	8.4 ± 3.9
	Active HF	33.7 ± 14.1	9.1 ± 6.2	6.8 ± 4.1
	Not active	42.3 ± 21.0	12.8 ± 8.0	9.8 ± 5.2
***p*** **-value**	Active LF–Active HF	*p* = 0.001 *	*p* < 0.001 *	*p* < 0.001 *
	Active LF–Not active	*p* = 0.006 *	*p* = 0.002 *	*p* = 0.007 *
	Active HF–Not active	*p* < 0.001 *	*p* < 0.001 *	*p* < 0.001 *
**Training experience**	0 years	42.3 ± 21.0	12.8 ± 8.0	9.8 ± 5.2
	1–3 years	37.6 ± 15.8	11.2 ± 6.7	8.5 ± 4.5
	4–7 years	36.6 ± 17.5	9.9 ± 5.8	7.7 ± 3.8
	More than 7 years	33.3 ± 13.2	9.0 ± 6.0	7.0 ± 4.0
***p*** **-value**	0 years–1–3 years	*p* = 0.250	*p* = 0.103	*p* = 0.002 *
	0 years–4–7 years	*p* = 0.015 *	*p* = 0.002 *	*p* < 0.001 *
	0 years–more than 7 years	*p* < 0.001 *	*p* < 0.001 *	*p* < 0.001 *
	1–3 years–4–7 years	*p* = 0.137	0.0113	0.205
	1–3 years–more than 7 years	*p* < 0.001 *	*p* < 0.001 *	*p* < 0.001 *
	4–7 years–more than 7 years	*p* = 0.028 *	*p* = 0.06	*p* = 0.003 *
**COVID-19 symptoms duration**	No symptoms	31.6 ± 12.3	6.7 ± 4.7	6.5 ± 3.1
	Few days	33.5 ± 13.7	8.5 ± 5.1	6.9 ± 3.3
	1 month	44.5 ± 20.4	15.2 ± 6.9	10.3 ± 5.1
	Some months	50.5 ± 21.9	18.4 ± 6.6	12.8 ± 5.7
***p*** **-value**	No symptoms–Few days	*p* = 0.001 *	*p* < 0.001 *	*p* = 0.062
	No symptoms–1 month	*p* < 0.001 *	*p* < 0.001 *	*p* < 0.001 *
	No symptoms–Some months	*p* < 0.001 *	*p* < 0.001 *	*p* < 0.001 *
	Few days–1 months	*p* < 0.001 *	*p* < 0.001 *	*p* < 0.001 *
	Few days–Some months	*p* < 0.001 *	*p* < 0.001 *	*p* < 0.001 *
	1 month–Some months	*p* = 0.005 *	*p* < 0.001 *	*p* < 0.001 *
**(b)**				
**Categories**	**Groups**	**FSMC Tot**	**FSCM Cogn**	**FSMC Phys**
**Gender**	Male	38.5 ± 22.1	19.5 ± 11.0	20.9 ± 11.1
	Female	48.4 ± 24.8	24.3 ± 12.5	25.8 ± 12.3
***p*** **-value**	Male–Female	*p* < 0.001 *	*p* < 0.001 *	*p* < 0.001 *
**Age**	Under 55	47.5 ± 24.5	23.9 ± 12.4	25.3 ± 12.1
	Over 55	41.9 ± 23.8	21.2 ± 11.9	22.6 ± 12.0
***p*** **-value**	Under 55–Over 55	*p* < 0.001 *	*p* < 0.001 *	*p* < 0.001 *
**Primary metabolic demand**	Aerobic	41.4 ± 22.1	20.9 ± 11.1	22.3 ± 11.0
	Mixed	41.9 ± 23.5	21.1 ± 11.7	22.5 ± 11.5
	Not active	51.5 ± 26.1	25.9 ± 13.1	27.4 ± 13.0
***p*** **-value**	Aerobic–Mixed	*p* = 0.923	*p* = 0.924	*p* = 0.851
	Aerobic–Not active	*p* < 0.001 *	*p* < 0.001 *	*p* < 0.001 *
	Mixed–Not active	*p* < 0.001 *	*p* < 0.001 *	*p* < 0.001 *
**Physical activity frequency**	Active LF	45.0 ± 23.5	22.8 ± 12.1	24.3 ± 11.5
	Active HF	39.0 ± 21.6	19.7 ± 10.6	21.0 ± 10.7
	Not active	51.5 ± 26.1	25.9 ± 13.1	27.4 ± 13.0
***p*** **-value**	Active LF–Active HF	*p* < 0.001 *	*p* < 0.001 *	*p* < 0.001 *
	Active LF–Not active	*p* = 0.007 *	*p* = 0.005 *	*p* = 0.005 *
	Active HF–Not active	*p* < 0.001 *	*p* < 0.001 *	*p* < 0.001 *
**Training experience**	0 years	51.5 ± 26.1	25.9 ± 13.1	27.4 ± 13.0
	1–3 years	49.1 ± 23.4	24.6 ± 12.0	26.1 ± 11.3
	4–7 years	41.1 ± 22.1	20.8 ± 11.5	22.2 ± 10.8
	More than 7 years	38.8 ± 21.7	19.6 ± 10.7	21.0 ± 10.9
***p*** **-value**	0 years–1–3 years	*p* = 0.561	*p* = 0.513	*p* = 0.375
	0 years–4–7 years	*p* < 0.001 *	*p* < 0.001 *	*p* < 0.001 *
	0 years–more than 7 years	*p* < 0.001 *	*p* < 0.001 *	*p* < 0.001 *
	1–3 years–4–7 years	*p* = 0.003 *	*p* = 0.002 *	*p* = 0.004 *
	1–3 years–more than 7 years	*p* < 0.001 *	*p* < 0.001 *	*p* < 0.001 *
	4–7 years–more than 7 years	*p* = 0.221	*p* = 0.330	*p* = 0.209
**COVID-19 symptoms duration**	No symptoms	33.2 ± 17.8	17.1 ± 8.7	18.1 ± 9.2
	Few days	37.8 ± 19.3	19.1 ± 9.8	20.7 ± 9.9
	1 month	58.5 ± 24.1	29.1 ± 12.4	31.1 ± 11.7
	Some months	68.3 ± 24.3	34.3 ± 12.4	35.2 ± 11.5
***p*** **-value**	No symptoms–Few days	*p* < 0.001 *	*p* = 0.001 *	*p* < 0.001 *
	No symptoms–1 month	*p* < 0.001 *	*p* < 0.001 *	*p* < 0.001 *
	No symptoms–Some months	*p* < 0.001 *	*p* < 0.001 *	*p* < 0.001 *
	Few days–1 months	*p* < 0.001 *	*p* < 0.001 *	*p* < 0.001 *
	Few days–Some months	*p* < 0.001 *	*p* < 0.001 *	*p* < 0.001 *
	1 month–Some months	*p* < 0.001 *	*p* < 0.001 *	*p* = 0.001 *

* = significant differences; LF = low frequency (less than three times per week of PA); HF = high frequency (more than three times per week of PA); SOBQ = Shortness of Breath Questionnaire; PHQ-9 = Patient Health Questionnaire-9 item; GAD-7 = Generalized Anxiety Disorder 7-item; PASE = Physical Activity Scale for Elderly. FSMC Tot = Fatigue Scale for Motor and Cognitive Functions Total score; FSMC Cogn = Fatigue Scale for Motor and Cognitive Functions Cognitive score; FSMC Phys = Fatigue Scale for Motor and Cognitive Functions Physical score.

**Table 3:** Correlation between questionnaire, training experience, and PASE.

Variable		SOBQ	PHQ-9	GAD-7	FSMC Tot	FSMC Cogn	FSMC Phys	PASE	Training Experience
**Training experience**	*rho*	−0.21 ^#^	−0.23 ^#^	−0.32 ^§^	−0.22 ^#^	−0.23 ^#^	−0.22 ^#^	0.16 ^#^	-
	*p*-value	<0.001 *	<0.001 *	<0.001 *	<0.001 *	<0.001 *	<0.001 *	<0.001 *	
**PASE**	*rho*	−0.42	−0.41	−0.61	−0.11	−0.22	−0.13	-	0.16 ^#^
	*p*-value	0.183	0.197	0.052	0.732	0.495	0.687		<0.001 *

* = denotes significant correlation; ^#^ = denotes weak correlation; ^§^ = denotes moderate correlation; SOBQ = Shortness of Breath Questionnaire; PHQ-9 = Patient Health Questionnaire-9 item; GAD-7 = Generalized Anxiety Disorder 7-item; FSMC Tot = Fatigue Scale for Motor and Cognitive Functions Total score; FSMC Cogn = Fatigue Scale for Motor and Cognitive Functions Cognitive score; FSMC Phys = Fatigue Scale for Motor and Cognitive Functions Physical score; PASE = Physical Activity Scale for Elderly.

**Table 4:** Predictors of all symptoms assessed by multiple regression model.

Categories	R	R^2^	F	B	SE	t	VIF	*p*-Value
**SOBQ**	0.198	0.039	20.383					
**PASE**				−0.007	0.011	−0.0635	1.000	0.526
**Training experience**				−0.283	0.046	−6.154		<0.001 *
**PHQ-9**	0.222	0.049	26.061					
**PASE**				−0.003	0.004	−0.662	1.000	0.508
**Training experience**				−0.128	0.018	−6.974		<0.001 *
**GAD-7**	0.238	0.057	29.984					
**PASE**				0.001	0.003	0.345	1.000	0.730
**Training experience**				−0.92	0.012	−7.683		<0.001 *
**FSMC Tot**	0.205	0.042	22.004					
**PASE**				0.007	0.015	0.445	1.000	0.656
**Training experience**				−0.418	0.063	−6.599		<0.001 *
**FSMC Cogn**	0.204	0.042	21.815					
**PASE**				0.003	0.008	0.457	1.000	0.647
**Training experience**				−0.209	0.32	−6.572		<0.001 *
**FSMC Phys**	0.203	0.041	21.462					
**PASE**				0.003	0.008	0.351	1.000	0.726
**Training experience**				−0.205	0.032	−6.507		<0.001 *

R^2^ = increase in the explained variance of the model; * = significance level of the increased R^2^; VIF = variance inflation factor; SOBQ = Shortness of Breath Questionnaire; PHQ-9 = Patient Health Questionnaire-9 item; GAD-7 = Generalized Anxiety Disorder 7-item; PASE = Physical Activity Scale for Elderly.

## Data Availability

Data are available upon request from the corresponding author, due to ethical restriction.
